# Phenolic Compounds in Mesoamerican Fruits—Characterization, Health Potential and Processing with Innovative Technologies

**DOI:** 10.3390/ijms21218357

**Published:** 2020-11-07

**Authors:** Andrea Gómez-Maqueo, Zamantha Escobedo-Avellaneda, Jorge Welti-Chanes

**Affiliations:** 1 Food Structure Team, Clinical Nutrition Research Center, Singapore Institute of Food and Biotechnology Innovation, Agency for Science, Research and Technology, 14 Medical Drive #07-02, MD 6 Building, Yong Loo Lin School of Medicine, Singapore 117599, Singapore; andrea_gomez_maqueo_cerecer@sifbi.a-star.edu.sg; 2 Escuela de Ingeniería y Ciencias, Tecnologico de Monterrey, Av. Eugenio Garza Sada 2501 Sur, Col. Tecnológico, Monterrey 64849, Nuevo León, Mexico

**Keywords:** phenolic compounds, fruits, bioactivity, high hydrostatic pressure, pulsed electric fields, nonthermal, Mexico, Mesoamerica

## Abstract

Diets rich in phenolic compounds have been associated to reducing the risk of metabolic syndrome and its derived disorders. Fruits are healthy components of the human diet because of their vitamin, mineral, fiber and phenolic profile. However, they have a short shelf-life which is limited by microbiological growth and enzymatic activity. Innovative preservation methods such as high hydrostatic pressure, pulsed electric fields, ultrasound, microwave, cold plasma and ultraviolet light have become popular for the processing of fruits because they can preserve nutritional quality. In this review, the phenolic profile and health potential of 38 Mesoamerican fruits were assessed. Phenolic compounds were classified based on their contribution to the diet as flavonoids, phenolic acids, tannin, lignins and stilbenoids. Due to this composition, fruits showed a wide range of bioactivities which included anti-inflammatory, anti-diabetic, anti-hypertensive and anti-obesity activities, among others. Phenolic content in fruits submitted to innovative food processing technologies depended on parameters such as enzymatic activity, antioxidant capacity, microstructure integrity and cell viability. Innovative technologies could increase phenolic content while assuring microbiological safety by (i) promoting the release of bound phenolic compounds during processing and (ii) inducing the synthesis of phenolic compounds by activation of phenylpropanoid pathway during storage.

## 1. Introduction

A significant event in human history was the transformation from a hunting-gathering economy to an agricultural economy. This transformation occurred independently in at least six regions of the world, mainly in tropical and subtropical areas with high biological and cultural diversity [1]. Mesoamerica is considered one of the world’s primary centers of domestication where species such as maize (*Zea mays* L.), beans (*Phaseolus* spp.) and squash (*Cucurbita* spp.) were domesticated and integrated into a multi-crop system. Mesoamerica is an historical region in North America which extends from the center of Mexico, through Belize, Guatemala, El Salvador, Honduras, Nicaragua and to the north of Costa Rica. From 2500 BC to AD 1521 Mesoamerica was a cultural area defined by its indigenous cultures such as the Aztecs, Mayas, Olmecs, among many others. Mesoamerica is an important center of genetic diversity and is recognized because of its role in plant domestication.

Fruits have become a fundamental part of the human diet and are of great relevance from a nutritional and economic perspective and they are rich sources of vitamins, minerals and dietary fiber. From a nutritional point of view, interest in fruits has increased in the last years because of their association with the prevention of chronic diseases attributed to their antioxidants such as phenolic compounds. Epidemiological studies have shown that daily intake of plant-derived foods plays a role in reducing the risk of some types of cancer, cardiovascular diseases and diabetes [2].

The domestication of fruits by Mesoamerican cultures have helped shape the face of today’s agriculture and cuisine in all the world. Nowadays, Mesoamerican fruits such as papaya (*Carica papaya* L.), tomato (*Solanum lycopersicum* L.), avocado (*Persea americana* Mill.), bell pepper (*Capsicum annuum* L.), zucchini (*Cucurbita pepo* L.), dragon fruit (*Hylocereus undatus* (Haw.) Britton et Rose) and pitaya (*Stenocereus stellatus* (Pfeiff.) Riccob) are cultivated and consumed on a worldwide scale. India, Brazil and Indonesia are currently the world-leading producers of papaya. China is currently the world’s largest producer of Mesoamerican fruits such as tomato, bell pepper and cainito (*Chrysophyllum cainito* L.). Meanwhile, Vietnam is one of the most important producers of dragon fruit and cashew (*Anacardium occidentale* L.), worldwide. Japan, USA and France are currently world-leading producers of zucchini. In addition, Mexico is the world’s largest producer of avocado, bell pepper and prickly pear (*Opuntia ficus-indica* L. Mill.) and maintains a diverse local production of lesser known Mesoamerican fruits such as chagalapoli (*Ardisia compressa* Kunth), nance (*Byrsonima crassifolia* (L.) Kunth), cactus berry (*Myrtillocactus geometrizans*), xoconostle (*Opuntia joconostle* Web.) and capulin (*Prunus serotina* Ehrh.).

Other fruits that are native to and/or were domesticated in the Mesoamerican region and that are included in this review are namely, cherimoya (*Annona cherimola* Mill.); annona (*Annona diversifolia* Saff.); soursop (*Annona muricate* L.); custard apple (*Annona reticulata* L.); sugar apple (*Annona squamosa* L.); jalapeño pepper, poblano pepper, serrano pepper, Yahualica pepper, chilaca pepper (*Capsicum annuum* L.); habanero pepper (*Capsicum chinense* Jacq.); manzano pepper (*Capsicum pubescens* Ruiz et Pav.); Mexican hawthorn (*Crataegus mexicana* Moc. et Sessé); black sapote (*Diospyros digyna* Jacq.); sapodilla (*Manilkara zapota* (L.) P. Royen); mamoncillo (*Melicoccus bijugatus* Jacq.); tomatillo (*Physalis philadelphica* Lam.); canistel (*Pouteria campechiana* (Kunth) Baehni); mamey (*Pouteria sapota* (Jacq.) H.E. Moore et Stearn); guava (*Psidium guajava* L.), squash (*Sechium edule* (Jacq.) Swartz); yellow mombin (*Spondias mombin* L.); and red mombin (*Spondias purpurea* L.).

Fruits are important sources of phenolic compounds such as phenolic acids, flavonoids, anthocyanins, tannins, lignins and stilbenoids that have shown a diversity of health benefits related to metabolic syndrome [3]. Much work has been carried out internationally regarding the characterization of specific families of phenolic compounds in the 38 fruits of Mesoamerican origin included in this review. By integrating the data reported in 105 studies, we provide an integral view of the complete phenolic profile that has been studied until now, as well as the bioactivity of mentioned fruits which is related to reducing the risk of obesity and metabolic syndrome-derived disorders.

Despite their healthy attributes, fruits products are perishable systems with a limited shelf-life due to the microbiological, biochemical and enzymatic reactions taking place during storage. Fruit products such as fresh-cut fruit, juices, beverages, nectar, puree and jams are widely consumed in today’s market where consumers are demanding healthy processed foods. Food preservation technologies are becoming more sophisticated in response to the growing demand for food quality, extended shelf-life and high-quality products with nutritional and functional characteristics [4]. The requirements of the preservation of foods have gradually changed throughout the years from seeking innocuous products with a long shelf life to include a high content of nutrients and antioxidants [5].

Thermal treatments such as pasteurization sterilization and concentration, are traditionally used to extend the shelf life of fruits and fruit-based-products. Nevertheless, thermal processing technologies may reduce the overall quality of the product including nutritional and sensory changes.

Due to the high consumer demand for minimally processed products with high nutritional and sensory quality, alternative preservation methods have gained relevance [4]. These include high hydrostatic pressure (HHP), pulsed electric fields (PEF), ultrasound (US), microwave (MW), cold plasma (CP) and ultraviolet light (UV).

Numerous international studies have reported the nutritional, phenolic composition and health benefits of some fruits of Mesoamerican origin. However, the extended and detailed phenolic composition in these fruits is yet to be compiled and analyzed. In addition, the association of phenolic compounds in these fruits with health benefits related to lowering the risk of metabolic syndrome requires further attention. Furthermore, the effects of innovative technologies on phenolic compounds in Mesoamerican fruits has been only reported for selected cultivars and the mechanisms for the observed changes in phenolic content often remains unexplained. This paper aims to provide a detailed review of the micronutrient composition, phenolic profile and health benefits of Mesoamerican fruits, as well as a critical overview of the effects of innovative food processing technologies on phenolic content after treatment and during storage as well as the mechanisms behind each technology. By recommending technologies or treatment intensities that could assure microbiological safety in fruit products while preserving or increasing phenolic content, we expect to contribute to future production of health-promoting fruit products.

## 2. Nutritional Composition, Phenolic Compounds and Health Potential of Mesoamerican Fruits

### 2.1. Description and Geographical Region

Mesoamerica is a cultural region that influenced Mexican cultures in the pre-Columbian era and is considered an important center of genetic diversity. The scientific name, description and geographical origin of the 38 Mesoamerican fruits included in this review is shown in Table 1. These include 11 tropical fruits, 5 fruits from cactus, 8 members of the *Capsicum annuum* L. specie (peppers), 3 that are usually classified as culinary vegetables (tomato, squash and zucchini), among others.

The domestication of the zucchini in Mexico is considered the first domestication of plants in America (10,000) years ago [18]. Other fruits that are native to the region of Mexico include chagalapoli, peppers of the *Capsicum annum* L. species, Mexican hawthorn, dragon fruit, sapodilla, cactus berry, prickly pear, canistel, capulin and avocado. Tomatoes, despite being originated in ancestral Peru-Ecuador, were also domesticated in the region of Mexico from where their cultivated forms were later disseminated [30]. Avocados are wild progenitors of eastern and central highlands of Mexico through Guatemala to the Pacific coast of Central America and were domesticated in pre-Hispanic Mexico. In other terms, cactus fruits originated from central and southern Mexico where they were domesticated and include prickly pears, cactus berries, dragon fruit, pitaya and sour prickly pears (xoconostle).

Tropical fruits from the *Annonaceae* family such as cherimoya, annona, soursop, custard apple and sugar apple are native to Central America and northern South America [7]. Similarly, cashew apple, nance, cainito and mamoncillo are also native to southern Mesoamerica and northern South America (Table 1).

### 2.2. Macronutrient Composition

The macronutrient composition of Mesoamerica fruits is shown in Table 2. Fruits such as peppers, zucchini, tomatillo, squash and tomato possess a high water content (90–95%). Meanwhile, the chagalapoli has a considerably higher protein content (8.6 g protein/100 g fresh fruit) the other fruits which range from 2.7 to 0.2 g protein/100 g fresh fruit. Regarding the fat content of fruits, avocado has a 15% fat content which consists of monounsaturated fatty acids (71%), polyunsaturated fatty acids (13%) and saturated fatty acids (16%), which have been associated with healthy blood lipid profiles and with better bioavailability of fat soluble vitamins and carotenoids [32].

In other terms, fruits such as mamey, custard apple, sugar apple, canistel and Mexican hawthorn possess a higher carbohydrate content (24–36%). Dietary fiber content is an important fraction of total carbohydrates because it is associated with a reduced risk of diabetes, heart disease and some types of cancer [33,34,35]. The American Dietetic Association recommends 14 g of dietary fiber per 1000 kcal or 25 and 38 g for adult women and adult men, respectively [33]. Of the Mesoamerican fruits described in Table 2, nance, avocado, guava, mamey, black sapote and sapodilla all contain high dietary fiber content were a consumption of 100 g could account for 21–31% and 14–20% of the total dietary intake for adult woman and men, respectively.

### 2.3. Micronutrient Composition

The mineral and vitamin content of Mesoamerican fruits is shown in Table 3. Potassium was the most abundant mineral in Mesoamerican fruits and ranged from 125 to 660 mg/100 g contributing from 3.5 to 19% of the daily recommended intake for adults [44]. Fruits that are rich in calcium include nance, Mexican hawthorn and prickly pears (45–56 mg/100 g).

Regarding vitamins, fruits such as nance, bell pepper, jalapeño pepper, poblano pepper, habanero pepper, chilaca pepper, Mexican hawthorn, papaya and guava are rich sources of vitamin C showing from 1.4 to 5 times higher content than orange juice. Foods such as avocado and squash contain about 42% and 48%, respectively, of the total folate content of spinach which is a product known for its high content of this vitamin. In other terms, fruits such as nance, bell pepper, jalapeño pepper, serrano pepper, papaya and tomato contained from 6% to 9% the retinol activity equivalents of carrots [37].

### 2.4. Phenolic Compounds

Phenolic compounds are secondary metabolites that are widely distributed in nature and influence the taste, flavor and appearance of vegetable foods. They consist of an aromatic ring with one or more hydroxyl groups and their structures vary from a simple molecule to a high molecular mass polymer. Phenolic compounds can be classified based on their contribution to the human diet [3]. Mentioned classification consists of phenolic acids (hydroxycinnamic and hydroxybenzoic acids) which represent 1/3 of the daily intake, flavonoids (anthocyanins, flavonols, flavanols, flavones, flavanones, isoflavones and proanthocyanidins) which contribute to 2/3 of the daily intake and others (tannins, lignans and stilbenes) which contribute in minimal amounts to the regular intake of phenolic compounds.

The information regarding the characterization of phenolic compounds in the fruits of Mesoamerican origin was reviewed in 63 international publications. Most publications relied on advanced chromatography techniques such as high performance liquid chromatography (HPLC) with diode array detector (DAD) or electrospray-Quadrupole-Time of Flight tandem mass spectrometry detector (ESI-Q-Tof) and ultra-performance liquid chromatography with electrospray ionization tandem mass spectrometry (UPLC-ESI-MS/MS) techniques for the characterization of phenolic compounds of a specific family. By compiling the detailed information from mentioned studies, the complete phenolic profile consisting of phenolic acids, flavonoids, tannins, lignans and stilbenes is shown in Table 4.

The total phenolic content of fruits described in Table 4 were mainly quantified colorimetrically by the Folin-Ciocalteu method. Although advanced chromatography techniques can also provide total phenolic content, these are determined by the sum of the identified and quantified compounds which are sometimes limited to a particular family of phenolic compounds. In Table 4, total phenolic contents ranged from 11 to 1056 mg/100 g fresh weight. The fruits with the highest phenolic content were namely, cactus berry, chagalapoli, chilaca pepper and zucchini. The high variability in phenolic content reported by various authors could be due to different extraction and analysis methods. In addition, different maturity stages and differences among the cultivars could also play a role in mentioned variability.

#### 2.4.1. Phenolic Acids

Phenolic acids are made up of two carbon frameworks. The two families of phenolic acids, hydroxycinnamic and hydroxybenzoic acids, have a different position hydroxyl groups on the aromatic ring. Hydroxybenzoic acids are less common than hydroxycinnamic acids but can form tannins (gallotannins and ellagitannins) and act as intermediates in lignin biosynthesis [152].

According to this revision, the profile in phenolic acids of all fruits (except annona and yellow mombin) has been reported (Table 4). Most fruits showed the presence of phenolic acids. p-coumaric, caffeic, ferulic and sinapic acid (hydroxycinnamic acids) and p-hydroxybenzoic, vanillic, syringic and protocatechuic acids (hydroxybenzoic acids). Caffeic acid is usually one of the most abundant phenolic acids in fruits representing up to 75% to 100% of the total hydroxycinnamic acid content [153,154]. Of the 38 fruits included in this review, 63%, 38%, 35% and 31% of them contained caffeic, p-coumaric, ferulic and sinapic acid, respectively. Meanwhile, p-hydroxybenzoic, vanillic, syringic and protocatechuic acids were present in 40%, 19%, 6% and 38% of the fruits of Table 4, respectively. The presence of phenolic acids in fruits is related to increases in bile secretion, reduction of blood cholesterol, lipid levels, as well as antimicrobial activity as major health benefits.

#### 2.4.2. Flavonoids

The major flavonoids for all fruits included in this review were reported (except mamoncillo). Flavonoids are the most widely distributed phenolic compounds in foods as they make up 2/3 of the dietary intake. They are made up by phenylbenzopyran that includes a C_15_ (C_6_-C_3_ C_6_) skeleton joined to a chroman ring. Flavonoids can be further subcategorized according to their structure as flavonols, flavanones, flavones, anthocyanins, flavonols and isoflavones [3].

Flavanols can be found as catechins (monomers) or as proanthocyanidins (polymers). The Mesoamerican fruits cherimoya, green and purple sugar apple, chagalapoli, green, yellow and red nance, green and purple cainito, black zapote, sapodilla, cactus berry, sour prickly pear, avocado, tomatillo, canistel, mamey, capulin, guava and yellow mombin contained catechins. Meanwhile, their oligo- or polymeric forms (proanthocyanidins) were reported in studies from avocado, mamey, capulin and guava (Table 4).

Flavanones can be found in fruits as aglycones (i.e., naringenin, hesperetin and eriodictyol) but more often are found as glycosylated compounds, either as neohesperidosides presenting a bitter taste (naringin) or as rutinosides without flavor [154]. In Table 4, naringenin was reported in jalapeño, poblano, serrano, Yahualica, chilaca, habanero and manzano peppers, as well as in dragon fruit (pitahaya), pitaya and tomato. Meanwhile, eriodictyol was present in dragon fruit (pitahaya) and pitaya.

Flavonols are the most abundant flavonoids in foods found as glycosylated compounds associated to glucose or rhamnose. Kaempferol and quercetin are common types of flavonols. In Mesoamerican fruits, kaempferol is present in soursop, chagalapoli, nance, *Capsicum annuum* L., zucchini, dragon fruit (pitahaya), pitaya, picky pear, avocado, capulin, guava, tomato and mombin; while quercetin is present in 67% of the Mesoamerican fruits of Table 4.

Flavones are typically less abundant in foods, where luteolin and apigenin are examples of flavones that can be mainly found as glycoside forms. In Table 4, luteolin was present in bell pepper, *Capsicum annuum* L., zucchini and squash, while apigenin was reported in *Capsicum annuum* L., papaya, black zapote, squash and tomato (Table 4).

Anthocyanins are colored compounds that refer to the glycoside or acyl-glycoside of anthocyanidins. They are found in high quantities in berries (black raspberries, elderberries, chokeberry and blackberries) and are responsible for their characteristic color. Anthocyanins reported in Table 4 were found in colored fruits such as cashew apple, chagalapoli, capulin, nance and in purple verities of tomatillo (Table 4). Their main biological activities include anti-inflammatory, antioxidant and chemoprotective activity. Cyanidin is the most common anthocyanin, followed by delphinidin, malvidin and peonidin [155].

#### 2.4.3. Tannins

Tannins are groups of phenylpropanoid compounds that are condensed to polymers of different lengths which can be classified as proanthocyanidins, hydrolysable tannins, phlorotannins and complex tannins based on their chemical structures and constitutive monomers. Proanthocyanidins can further be subclassified as condensed tannins and are the polymerized product of flavan-3-ols (catechins) and flavan-3,4-diols or both [156,157]. Typically, fruits such as berries are the major sources of proanthocyanidins in the human diet. In Table 4, cactus berry, avocado, mamey, capulin and guava had proanthocyanidins content. In other terms hydrolysable tannins refer to gallotannins and ellagitannins and upon hydrolysis, yield gallic acid or ellagic acid, respectively. In Table 4, mentioned hydrolysable tannins were reported in fruits such as red and yellow cashew apple, cherimoya, green and purple sugar apple, chagalapoli, guava, green and purple caimito, Mexican hawthorn, zucchini, black zapote, dragon fruit (pitahaya), cactus berry and canistel.

#### 2.4.4. Lignans

Lignans were reported in green, red and yellow bell pepper and *Capsicum annuum* L. (Table 4). Mentioned compounds are made up of two phenylpropane units and are one of the major sources of phytoestrogens in plants. They have significantly lower contribution to the human diet compared to flavonoids and phenolic acids [157]. They can be transformed by the intestinal microbiota to enterolignans which contribute to reducing the risk of certain cancers and cardiovascular diseases [3].

#### 2.4.5. Stilbenes

Like lignans, stilbenes also contribute little to the dietary intake of phenolic compounds. Resveratrol is the most widely studied stilbene which has shown antioxidant, anti-inflammatory, estrogenic, cardioprotective, anti-tumor and anti-viral activities [158]. Resveratrol, besides being found in grapes and grape derived products, can also be found in the fruits such as mamoncillo (Table 4).

### 2.5. Health Benefits of Mesoamerican Fruits

The study of bioactive compounds such as phenolics in fruits has become of great interest from biological, medical, and nutritional points of view because they contribute to the reduction of risk factors of diseases related to metabolic syndrome. The health potential of fruits is mostly studied by assessing the bioactivity of a certain extract that contains health-promoting constituents such as phenolic compounds. In this review, 42 articles were identified because they focus on the health potential of phenolic extracts of the edible fraction of fruits of Mesoamerican origin (Table 4). Extensive evidence has demonstrated that dietary phenolic compounds can act as antioxidant and anti-inflammatory agents by increasing thermogenesis and energy expenditure and reducing oxidative stress [159]. Most of the studies included in this review are related to reducing the risk of obesity and metabolic syndrome-related disorders. Currently, the health potential of subproducts of the fruit processing industry or non-edible fractions has gained great interest but is outside the scope of the present review.

In all fruits, the in vitro antioxidant capacity of hydrophilic extracts was reported. In vitro antioxidant activities from 3–17 µm Trolox Eq./100 g fresh weight (ABTS assay) were reported for cactus berry and guava; from 400–600 µm Trolox Eq./100 g fresh weight for sapodilla, black sapote, yellow bell pepper and mamey; from 600–700 µm Trolox Eq./100 g fresh weight for red cashew apple, yellow cashew apple, annona, custard apple, green sugar apple, purple sugar apple, green nance, red nance, yellow nance, red bell pepper, chilaca pepper, papaya, green canito, purple canito, mamoncillo, yellow mombin and red mombin; from 700–900 µm Trolox Eq./100 g fresh weight for cherimoya, green bell pepper, chilaca pepper and dragon fruit; and from 2000 to 6400 µm Trolox Eq./100 g fresh weight for chagalapoli, habanero type pepper and sour prickly pear (Table 4).

Regarding the in vitro studies, the most reported bioactivities were anticancer (10 fruits), anti-inflammatory (7 fruits) and antidiabetic (5 fruits). Mentioned in vitro bioactivity studies of phenolic-rich extracts showed anticancer activity for cherimoya, guava, tomato and black sapote; antidiabetic activity for annona; antibacterial and anti-inflammatory activities for peppers; antidiabetic, anti-inflammatory and anticancer activities for peppers; antiproliferative and anti-inflammatory activities for papaya; antihypertensive and anticancer activities for green and purple cainito; relaxant activity for Mexican hawthorn; anticancer and anti-inflammatory activities for pitahaya; anti-inflammatory, antidiabetic and anticancer activities for cactus berry; anticancer, anti-inflammatory and antidiabetic activity for green, purple, red and yellow prickly pear; anticancer, anti-inflammatory, antidiabetic activities for avocado; and anti-inflammatory activity for canistel (Table 4).

In other terms, the most reported in vivo studies included antidiabetic (10 fruits), antioxidant (6 fruits) and anti-obesity (4 fruits) activity. In vivo bioactivity studies of phenolic compounds in red and yellow cashew apples showed anti-diabetic, antioxidant, anti-obesity, anti-inflammatory and wound-healing activity. In green sugar apple, the presence of dietary phenolic compounds contributed to its antidiabetic and antioxidant activity. Meanwhile spicy peppers showed antidiabetic, hypocholesterolemic, cardioprotective and antiobesity activity. Phenolic extracts from green caimito showed antihypertensive and gastroprotective activity; antidiabetic, wound healing and antihypertensive activity for pitahaya; antitumor, anti-obesity and antidiabetic activity for sapodilla; antidiabetic and renal protective activity for cactus berry; and antidiabetic, antioxidant and kidney protective activity for prickly pears. Sour prickly pears (xoconostle) extracts have been studied regarding their antidiabetic and antioxidant activity. Furthermore, avocado extracts have shown anti-obesity and antidiabetic activity. Other fruits showed hepatoprotective and antioxidant activity for canistel; antihypertensive and vasorelaxant activity for capulin; antihypertensive, cardioprotective, antidiabetic, antiulcer and hepatic injury protective activity for squash; gastroprotective and ulcer healing activity for yellow mombin; and antioxidant activity for red mombin (Table 4).

## 3. Effects of Innovative Technologies on Phenolic Compounds in Fruits

Microbiological growth and enzymatic activity are the most important limiting factors in the shelf life of fruit-derived products. The main concerns in the fruit processing industry are related to contamination of yeasts (i.e., lactic and acetic acid bacteria), molds (i.e., *Byssochlamys, Talaromyces* and *Neosartorya*) and pathogenic microorganisms (i.e., *Escherichia coli* O157:H7, *Cryptosporidium parvum* and *Salmonella* spp. [160]. Furthermore, enzymes such as polyphenoloxidase (PPO), peroxidase (POD), pectin methylesterase (PME), lipoxygenase and catalase are the main enzymes responsible for fruit product quality changes (color, texture and flavor) during storage [161].

Innovative food processing technologies are currently being studied for (i) assuring food safety and stability, (ii) as pre-treatments in the manufacturing of food products to reduce energy consumption (i.e., prior to drying, freezing, extraction, distillation, etc.) and (iii) for the obtaining of extracts and nutraceutical development. The present review solely focuses on the use of innovative food processing technologies for the purpose of (i) assuring food safety and stability of fruits and fruit products. Furthermore, the study of these technologies should also include the effects on phenolic compounds and/or on parameters that affect the stability of phenolic compounds in foods (i.e., enzymatic activity, antioxidant capacity, microstructure integrity, color and cell viability).

In this review, 41 published articles with mentioned characteristics were identified. These included only 14 of the 38 fruits of Mesoamerican origin characterized previously. Mentioned fruits included custard apple, avocado, bell pepper, jalapeño pepper, cashew apple, guava, papaya, pitaya, prickly pear, sapodilla, soursop, mamey, dragon fruit and tomato. The fruit products studied in mentioned articles were juices, beverages, sliced fruits, pulps, purees, and jams.

### 3.1. High Hydrostic Pressure (HHP)

High Hydrostic Pressure (HHP) is a nonthermal preservation technology that can extend the shelf life of foods with little or no alterations to its sensory and nutritional characteristics. It is the most commercialized nonthermal technology with sales increasing annually by $10 billion USD and the number of high-pressure units growing exponentially at an annual rate [162]. HHP has shown a wide range of applications in the processing of fruits because these foods are highly susceptible to browning and color change by thermal treatments.

The processing of foods by HHP consists of introducing hermetically sealed products in a thermally insulated airtight vessel and subjecting them to high pressure (100–600 MPa). The pressure is transmitted inside the vessel instantaneously and uniformly by a liquid medium such as water. This uniform pressure (isostatic principle) causes microbiological death and enzymatic inactivation.

The effects of HHP on phenolic compounds and parameters related to their stability in fruits are shown in Table 5. The Mesoamerican fruits that have been treated with HHP include avocado, bell pepper, cashew apple, guaya puree, papaya, dragon fruit, prickly pears, sapodilla and tomato. The main mechanism which led to a higher phenolic content after processing with HHP was an increase in extractability of bound phenolic compounds.

Increases in total phenolic content were observed in HHP-treated cashew apple juice (25%), prickly pear beverages (35%), prickly pear slices (25–120%) and sapodilla jam (27%) (Table 5). In prickly pear slices, phenolic content increased with increasing pressure due to the modification of cell walls which promoted the release of cell-bound phenolic compounds, that where otherwise inaccessible [175].

HHP is the most effective technology to stabilize and extend the shelf-life of avocado pulp [164,166,179,180]. Until the last few decades, avocado derived products could not be successfully commercialized due to browning caused by the oxidative effect of PPO. Today, HHP-treated avocado paste has become one of the most successful products treated with this technology and has played a fundamental role in the boosting of commercial HHP units.

However, the degradation of phenolic compounds may also occur as shown for bell pepper treated at 100 MPa and accompanied by a lower antioxidant capacity and significant microstructural damage to cells [167]. Papaya and pitaya beverages processed at 400–600 MPa also showed lower phenolic content post treatment [170,171].

### 3.2. Pulsed Electric Fields (PEF)

Pulsed Electric Fields (PEF) is based on the application of external electric fields (1–50 kV/cm) for a short time (microseconds to milliseconds) to biological material and is based on the principle of electroporation.

Tomatoes were the only fruits of Mesoamerican origin that have been studied using PEF technology and report its effect on phenolic compounds (Table 6). PEF treatments in tomato fruits have a beneficial effect on phenolic content, particularly during storage, due to the activation of the phenylpropanoid pathway which leads to the synthesis of phenolic compounds as a mean of abiotic stress. In tomatoes, increases in phenolic compounds (19–57%) following PEF treatments have been observed after 24 h at 4 °C [181,182]. Mentioned studies showed that the increased number of pulses at 1.2 KV/cm promoted the synthesis of polyphenols in tomatoes as a stress response. This response was induced by the recognition of a stimulus at the cellular level (changes in electrical potential differences of the membranes) which influenced the voltage-gated ion channels and increased membrane permeability for Ca^2+^ at the cellular level. This was followed by a quick influx of Ca^2+^ through cation channels. Afterwards, Ca^2+^-dependent protein kinase (CDPK) phosphorylates PAL which regulates the phenylpropanoid metabolism that leads to the synthesis of new phenolic compounds [183]. CDPK can also increase the reactive oxygen species (ROS) which are endogenous signal components required for the synthesis of secondary metabolites [184].

In addition, it has been shown that (similar to HHP) PEF can promote the release of intracellularly bound phenolic compounds and contribute to a higher phenolic content as a secondary effect of electroporation [5]. Contrarily, high intensity PEF treatments for extended times tend to induce the degradation of phenolic compounds in fruits.

### 3.3. Ultrasound (US)

Ultrasound (US) for food processing uses inaudible sound waves at a frequency superior to 20 kHz. Ultrasound produces the cavitation of dissolved gas inside the liquid which causes the generation and evolution of microbubbles in a liquid medium. Once these microbubbles reach a critical size, they implode violently and return to their original size which causes the sudden release of all of the accumulated energy while instantly producing increases in local temperature (these are dissipated without substantially raising the temperature of the liquid) [185].

Ultrasound is used because of its effectiveness against undesired microorganisms found in liquid foods and can reach a 5-log reduction of some pathogens such as *E. coli* in fruit juices [186]. The energy that is released and the mechanical shock affects the microstructure of the cells or cell fragments in a liquid medium (i.e., puree, juice). Large cells are usually more sensitive to ultrasound than smaller ones and gram-negative bacteria are more susceptible to inactivation than Gram-positive bacteria.

Ultrasound treatments enhanced the extractability of phenolic compounds in Mesoamerican fruits such as cashew apple puree, custard apple juice, guava juice and prickly pear juice (Table 6). This effect was mainly attributed to the further rupture and modification of cell fragments in the processed fruits due to cavitation. Cavitation was also responsible for the increased water diffusivity in the samples which contributed to the extraction of phenolic compounds. In other terms, treating tomatoes at 45 kHz increased phenolic content 40% after 15 days of storage at 10 °C [187], which could be due to synthesis of these metabolites as a response to abiotic stress.

In the studies reporting the use of ultrasound in fruits of Mesoamerican origin, there were no reports on the degradation of phenolic compounds. However, a disadvantage of ultrasound treatments is that compared to HHP or PEF, a lower inactivation of microorganisms can be achieved. Furthermore, ultrasound treatments are most effective in juices and puree fruit products and less effective on solid foods.

### 3.4. Microwave (MW)

The use of microwaves as a food processing technique for microbial inactivation consists of two mechanisms: ionic interaction and dipolar rotation [188]. During microwave treatments, ionic polarization is induced by an electrical field. This electrical field causes ions in the food (mainly water) to move at an accelerated pace because of their inherent charge and collide with other ions. These molecular collisions convert kinetic energy into thermal energy.

For fruits, the use of microwaves has become important worldwide in the market of dehydrated products because of its ability to lower processing time. Similar to HHP, PEF and US, microwave treatments are also able to release bound phenolic compounds in fruits and hence increase antioxidant activity [189,190].

The fruits of Mesoamerican origin that have been processed with microwaves and report phenolic content or related parameters include avocado, guava, jalapeño pepper and mamey (Table 6). On one hand, avocado puree treated at 11 W/g resulted in a higher phenolic content (29%) post treatment and remained stable for 4 weeks stored at 4 °C [191] (Table 6). On the other hand, treating jalapeño peppers with ultrasound resulted in a degradation of phenolic compounds (21%) [192]. When compared to nonthermal technologies such as HHP and US, thermal treatments such as microwaves can have a more negative effect on phenolic content even with short processing times.

### 3.5. Cold Plasma (CP)

Cold plasma is one of the most recent nonthermal technologies for the preservation of foods, particularly as a sterilization treatment. Plasma is produced by applying electromagnetic fields to gas (usually O_2_ or N_2_) by generating a mixture of electrons, ions, atomic species, UV photons and charged particles that react with the food substrate. This can target microorganisms by releasing the stored energy [161]. The main parameters for processing with cold plasma are the gas feed, electric field, surrounding media and exposure time [210]. The cellular damages and surface modifications caused by plasma support its potential for increasing the extractability of hydrophilic compounds by decreasing the resistance to diffusion of internal molecules [211].

Cold plasma treatment resulted in a higher phenolic content in cashew apple juice, dragon fruit (pitahaya) and tomato beverage (Table 6). Treating cashew apple juice at 80 kHz resulted in a higher flavonoid (120%) and polyphenol content (128%) [205]. In dragon fruit treated at 60 kV, a higher total phenolic content of 28% was observed, accompanied by increases in gallic acid, protocatechuic acid and p-coumaric acid after 36 h storage at 15 °C [206]. Similarly, treating pitaya fruit with cold plasma at 60 kV, induced a similar response during storage as observed for PEF where there was an amplified signal role of ROS and the phenylpropanoid metabolism was activated leading to the synthesis of phenolic compounds. The use of cold plasma for the preservation of fruit products offers several advantages over other technologies such as it requires little energy and short treatment times, reactive gas species revert back to original gas within minutes to hours after treatment and it is a dry process that can be adaptable to a food manufacturing environment.

### 3.6. Ultraviolet Light (UV)

Ultraviolet light radiation (UV-C) consists of the applying nonionizing light (200–280 nm) to decontaminate the surface of fruits. The principle of UV-C decontamination is related to the damage of DNA. This kind of processing technology is easy to use, requires inexpensive equipment, does not leave residues and is lethal to most microorganisms (bacteria, viruses, protozoa, yeast, molds and algae [212,213].

**Figure 1:**
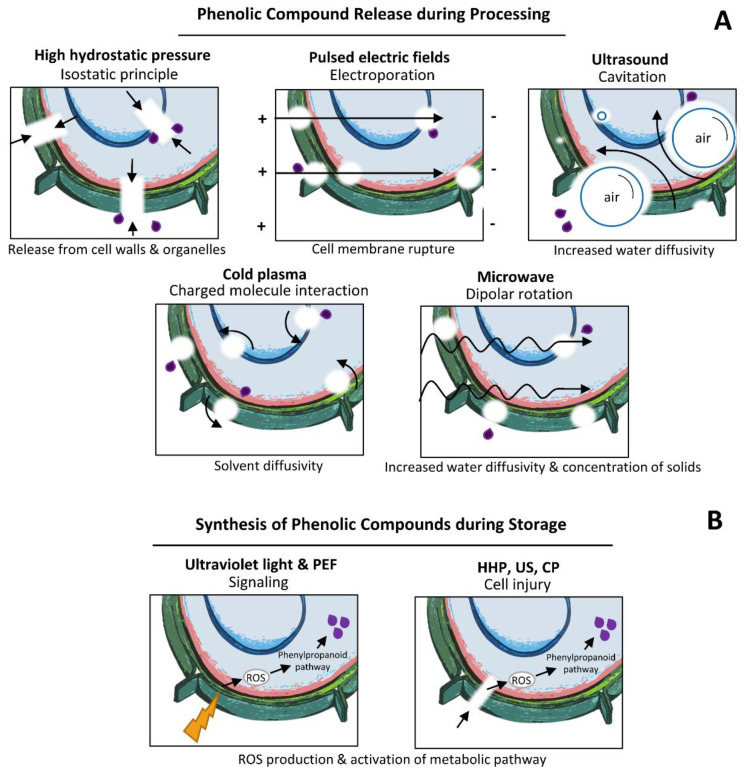
Mechanisms that drive increases in phenolic compound content in fruits treated with innovative technologies: (**A**) increases in the extractability of phenolic compounds and (**B**) synthesis of phenolic compounds during storage. Purple drops = phenolic compounds.

In other terms, ultraviolet light radiation (UV-B) (280–315 nm) is also used in fruits and vegetables as a mean of postharvest abiotic stress for the accumulation of health-promoting compounds such as phenolic compounds [214] (Figure 1). In Mesoamerican fruits such as dragon fruit (pitaya) juice (pitaya) and tomato beverages treated with UV-C light, no differences in phenolic content were found after treatment (Table 6). However, in prickly pear fruits treated with UV-B light at 6.4 W/m^2^ phenolic content increased 100% in whole pulp and 25% in wounded pulp after 24 h stored at 16 °C [209]. One of the disadvantages of this technology is that it may cause significant changes in the textural characteristics of the stressed tissue that may not be ideal for certain fruit-based products. However, the stressed tissue can be used as raw material to produce functional foods or for the further extraction and purification of compounds with applications in the pharmaceutical and dietary supplement industry.

### 3.7. Mechanisms of Innovative Technologies on Phenolic Compounds

As mentioned previously, innovative food processing technologies can affect phenolic compounds in fruit products. Excessive food processing intensities and time, as well as the partial inactivation of enzymes and food spoilage can lead to the degradation of most antioxidants. However, is applied properly, innovative food preservation technologies can simultaneously (i) assure microbiological safety while (ii) increasing/preserving phenolic content in foods. The main ways that phenolic compounds can increase during food processing or in storage conditions is by their (i) enhanced release (during processing) and (ii) synthesis (during storage).

Phenolic compounds can be found in soluble and insoluble-bound forms. Soluble phenolic compounds are localized in the vacuoles of plant cells where they are contained. Meanwhile, insoluble-bound phenolic compounds are attached to the cell wall matrix to macromolecules such as structural proteins, cellulose and pectin [215]. Phenolic content in foods can increase during food processing because of the release of insoluble-bound phenolic compounds from the cell walls (enhanced release/extractability). Innovative food processing technologies such as HHP, PEF, US, CP and MW can cause microstructural changes in vegetable cells that can promote the release of insoluble phenolic compounds from cell walls and macromolecules (Figure 1A).

During pressurization by HHP, vegetable tissues suffer structural modifications which favor a compact form. This causes changes in the fruit tissue on a cellular level such as changes in cell morphology, cell wall thickness and the rearrangement of cells [216] that promote the release of organelle-bound phenolic compounds. Meanwhile in PEF treatments, electroporation decreases the resistance to diffusion of phenolic compounds and promotes their extractability [178] as a mass transfer process. Ultrasound treatments at high intensities can disrupt cells, inhibit enzymes and enhance the yield of extraction of phenolic compounds by means of cavitation. The intensity required for cavitation to occur depends on the physical and chemical characteristics of the liquid media (vapor pressure, tensile strength, solid concentration and dissolved gas) [160]. In cold plasma, charged molecule interactions play a fundamental role in enhancing the extractability of phenolic compounds by increasing the diffusivity of the solvent. The cellular damages and surface modifications caused by plasma support its potential for increasing the extractability of hydrophilic compounds by decreasing the resistance to diffusion of internal molecules [211]. In microwave treatments, dipolar rotation can increase water diffusivity and the concentration of solids, hence, contributing to the release of phenolic compounds during processing. However, extended processing times can lead the degradation of these antioxidants.

In other terms, recent studies have shown that innovative food processing technologies could act as stress factors that may lead to a burst of reactive oxygen species (ROS) either by signaling (UV light and PEF) or by causing damage to vegetable cells (HHP, US, CP) (Figure 1B). These endogenous signal components are required for synthesis of secondary metabolites (i.e., phenolic compounds) as a defense response of plants to stress. This application of nonthermal processing technologies as abiotic stress elicitors to induce the accumulation of nutraceuticals in horticultural crops has been proposed as an innovative tool to obtain healthier fruits and vegetables [217].

## 4. Conclusions

This review provided a detailed compilation of the nutrient composition, phenolic profile and health benefits of 38 Mesoamerican fruits, as well a critical overview of the effects of innovative technologies on phenolic content.

We provided a complete overview on the phenolic composition in mentioned fruits by analyzing and selecting a total of 63 scientific articles. Phenolic compounds were classified from a nutritional point of view as phenolic acids (contribute to 1/3 of the diet), flavonoids (contribute to 2/3 of the diet) and tannins, lignins and stilbenoids (contribute in minor amounts to the diet). Cactus berry, chagalapoli, chilaca pepper and zucchini had the highest phenolic content.

In addition, the available information on the health potential of these fruits was compiled from 42 scientific articles that studied their phenolic-rich edible fractions. Most of the reported bioactivities in fruits were related to reducing the risk of disorders related to obesity and metabolic syndrome such as anti-inflammatory, anti-diabetic, anti-hypertensive, and anti-obesity activities.

Of the 38 fruits included in this review, the effects of innovative technologies on phenolic compounds and/or related parameters has only been studied in 14 fruits. A total of 41 studies were selected for comparing the different effects of these processing techniques on different fruit products such as juices, beverages, sliced fruits, pulps, purees, and jams. Phenolic content after food processing and during storage depended on parameters such as enzymatic activity, antioxidant capacity, microstructure integrity, color, and cell viability. Increases in phenolic content could be observed due to two main mechanisms (i) release during processing and (ii) synthesis during storage. HHP, PEF, US, CP, and MW could affect phenolic compounds release during processing by different mechanisms. UV and PEF could induce the synthesis of phenolic compounds by signaling. Similarly, HHP, US and CP could induce the synthesis of phenolic compounds by cell injury.

Fruits of Mesoamerican origin contain an abundant variety of phenolic compounds which contribute to their health potential. The adequate processing of fruits with innovative technologies is capable of simultaneously achieving food safety as well as preserving these antioxidant compounds. There is still a need for further research regarding the effects of innovative technologies on phenolic compounds in Mesoamerican fruits.

## Figures and Tables

**Table 1:** Scientific name, description and geographical origin of the Mesoamerican fruits included in this review.

	Scientific Name	Fruit Name (Spanish Name)	Native Regions
1	*Anacardium occidentale* L.	Cashew apple (Marañón)	Brazil and Central America [6].
2	*Annona cherimola* Mill.	Cherimoya (Chirimoya)	Mesoamerica [7].
3	*Annona diversifolia* Saff.	Annona (Ilama/Papausa)	Mesoamerica [8].
4	*Annona muricate* L.	Soursop (Guanábana)	Central America and northern South America [8].
5	*Annona reticulata* L.	Custard apple (Anona roja)	Mesoamerica (Guatemala and Belize) [8].
6	*Annona squamosa* L.	Sugar apple (Saramuyo)	Southeast Mexico [8].
7	*Ardisia compressa* Kunth	Chagalapoli	Tropical rain forests of Mexico [9].
8	*Byrsonima crassifolia* (L.) Kunth	Nance	Amazon region and tropical America [10].
9	*Capsicum annuum* L.	Bell pepper (Pimiento)	*Capsicum annuum* L. peppers (9–14): Domesticated species of *Capsicum annuum* var. Glabriusculum of Mesoamerican origin (Mexico) [11,12].
10	*Capsicum annuum* L.	Jalapeño pepper	
11	*Capsicum annuum* L.	Poblano pepper	
12	*Capsicum annuum* L.	Serrano pepper	
13	*Capsicum annuum* L.	Yahualica pepper	
14	*Capsicum annuum* L.	Chilaca pepper	
15	*Capsicum chinense* Jacq.	Habanero pepper	Amazon region (domesticated in Mesoamerica) [13].
16	*Capsicum pubescens* Ruiz et Pav.	Manzano pepper	Mesoamerica (Central and South America) [14].
17	*Carica papaya* L.	Papaya	Mesoamerica (Mexico) [15].
18	*Chrysophyllum cainito* L.	Cainito (Caimito)	Southern Mesoamerica (Panama) [16].
19	*Crataegus mexicana* Moc. et Sessé	Mexican hawthorn (Tejocote)	Mesoamerica (Mexico) [17].
20	*Cucurbita pepo* L.	Zucchini (Calabacita)	Mesoamerica (Mexico) [18].
21	*Diospyros digyna* Jacq.	Black sapote (Zapote negro)	Mesoamerica [19].
22	*Hylocereus undatus* (Haw.) Britton et Rose	Dragon fruit (Pitahaya)	Mesoamerica (central Mexico) [20].
23	*Manilkara zapota* (L.) P. Royen	Sapodilla (Chicozapote)	Mesoamerica (Mexico, Guatemala and Belize) [21].
24	*Melicoccus bijugatus* Jacq.	Mamoncillo (Guaya)	South America (Colombia and Venezuela) [22].
25	*Myrtillocactus geometrizans* (Mart. ex Pfeiff)	Cactus berry (Garambullo)	Arid and semiarid regions of Mexico [23].
26	*Opuntia ficus-indica* (L.) Mill.	Prickly pear (Tuna)	Mesoamerica (central and southern Mexico) [24].
27	*Opuntia joconostle* Web.	Sour prickly pear (Xoconostle)	Mesoamerica [25].
28	*Persea americana* Mill.	Avocado (Aguacate)	Mesoamerica (Mexico and Central America) [26].
29	*Physalis philadelphica* Lam.	Tomatillo	Mesoamerica (Mexico) [8].
30	*Pouteria campechiana* (Kunth) Baehni	Canistel (Zapote amarillo)	Mesoamérica (Bahamas, Belize, El Salvador, Guatemala and southern Mexico) [27].
31	*Pouteria sapota* (Jacq.) H.E. Moore et Stearn	Mamey	Mesoamerica [8].
32	*Prunus serotina* Ehrh.	Capulin	Mesoamerica (Mexico and Guatemala) [28].
33	*Psidium guajava* L.	Guava (Guayaba)	Mesoamerica [29].
34	*Sechium edule* (Jacq.) Swartz	Squash (Chayote)	Mesoamerica (southern Mexico and Guatemala) [8].
35	*Solanum lycopersicum* L.	Tomato (Jitomate)	Peru-Ecuador (domesticated in Mexico) [30].
36	*Spondias mombin* L.	Yellow mombin (Ciruela amarilla)	Mesoamerica [8].
37	*Spondias purpurea* L.	Red mombin (Ciruela roja)	Mesoamerica (Yucatán in Mexico) [8].
38	*Stenocereus stellatus* (Pfeiff.) Riccob.	Pitaya (Pitaya)	Mesoamérica (central Mexico) [31].

**Table 2:** Macronutrient composition (per 100 g) of Mesoamerican fruits.

	Fruit	Water	Protein	Fat	Carbohydrate ^1^	Fiber, Total Dietary	Ref.
		(g)	(g)	(g)	(g)	(g)	
1	Cashew apple	86.3	0.2	0.1	11.1	3.2	[36]
2	Cherimoya	79.4	1.6	0.7	17.7	3.0	[37]
3	White annona	79.6	1.1	0.3	13.6	4.4	[38]
	Pink annona	78.9	0.9	0.2	18.4	0.6	[37]
	Deep Pink annona	77.1	0.9	0.2	20.3	0.7	[37]
4	Soursop	81.2	1.0	0.3	16.8	3.3	[37]
5	Custard apple	71.5	1.7	0.6	25.2	2.4	[37]
6	Sugar apple	73.2	2.1	0.3	23.6	4.4	[37]
7	Chagalapoli	80.5	8.6	0.6	11.9	3.6	[39]
8	Nance	80.6	0.7	1.2	17.0	7.5	[37]
9	Bell pepper	93.3	0.9	0.2	5.1	1.8	[37]
10	Jalapeño pepper	91.7	0.9	0.4	6.5	2.8	[37]
11	Poblano pepper	93.9	0.9	0.2	4.6	1.7	[37]
12	Serrano pepper	90.3	1.7	0.4	6.7	3.7	[37]
14	Chilaca pepper	89.4	1.5	0.3	7.4	0.9 ^4^	[40]
15	Habanero pepper	91	2.3	0.8	3.6	1.6 ^4^	[40]
17	Papaya	88.1	0.5	0.3	10.8	1.7	[37]
18	Purple cainito	84.5	0.6	1.7	12.7	-	[40]
	White cainito	84.7	0.8	1.6	13.2	-	[40]
19	Mexican hawthorn	74.7	0.8	0.6	22.0	2.7 ^4^	[40]
20	Zucchini	92.7	2.7	0.4	3.1	1.1	[37]
21	Black sapote	83.6	0.6	1.1	14.5	5.3	[37]
22	Dragon fruit ^2^	82.3	1.4	0.1	13.6	2.1 ^4^	[40]
23	Sapodilla	78.0	0.4	1.1	20.0	5.3	[37]
26	Prickly pear	87.6	0.7	0.5	9.6	3.6	[37]
27	Sour prickly pears	87.6	1.1	0.1	6.7	4.0 ^4^	[41]
28	Avocado	73.2	2.0	14.7	8.5	6.7	[37]
29	Tomatillo	91.6	1.0	1.0	5.8	1.9	[37]
30	Canistel	60.6	2.0	0.5	35.9	-	[42]
31	Mamey	64.9	1.5	0.5	32.1	5.4	[37]
33	Guava	80.8	2.6	1.0	14.3	5.4	[37]
34	Squash	94.2	0.8	0.1	4.5	1.7	[37]
35	Tomato	94.8	1.2	0.2	3.2	0.9	[37]
36	Yellow mombin	70.4	1.4	0.1	26.7	-	[42]
37	Red mombin	76.2	0.9	0.1	22.0	-	[42]
38	White pitaya ^3^	86.6	1.1	0.5	9.8	1.6 ^4^	[43]
	Yellow pitaya	85.4	1.2	0.5	10.6	1.6 ^4^	[43]
	Purple pitaya	86.6	1.3	0.5	9.6	1.4 ^4^	[43]
	Red pitaya	86.4	1.3	0.4	9.8	1.6 ^4^	[43]

^1^ Calculated by difference for products obtained from USDA, 2020 [37]; ^2^ *Hylocereus undatus* (Haw.) Britton et Rose; ^3^ *Stenocereus stellatus* (Pfeiff.) Riccob; ^4^ Crude fiber.

**Table 3:** Micronutrient composition (per 100 g) of Mesoamerican fruits.

		Minerals										Vitamins								
	Fruit	Ca	Fe	Mg	P	K	Na	Zn	Cu	Mn	Se	Vit C	Thiamin (B1)	Riboflavin (B2)	Niacin (B3)	Pantothenic Acid (B5)	Pyridoxine (B6)	Folate, Total	Vit A ^3^	Ref.
		mg	mg	mg	mg	mg	mg	mg	mg	mg	µg	mg	mg	mg	mg	mg	mg	µg	µg	
1	Cashew apple	37	6.68	292	593	660	12	5.78	2.20	1.66	19.9	0.5	0.42	0.06	1.06	0.86	0.42	25.0	0.0	[36]
2	Cherimoya	10	0.27	17	26	287	7	0.16	0.07	0.09	-	12.6	0.10	0.13	0.64	0.35	0.26	23.0	0.0	[37]
3	White annona	0.9	-	8	-	348	2	0.13	-	-	-	2.4	-	-	-	-	-	-	-	[38]
	Pink annona	23	-	13	-	336	3	12.71	-	-	-	1.6	-	-	-	-	-	-	-	[37]
	Deep pink annona	14	-	14	-	347	3	14.01	-	-	-	1.5	-	-	-	-	-	-	-	[37]
4	Soursop	14	0.6	21	27	278	14	0.10	0.09	-	0.6	20.6	0.07	0.05	0.90	0.25	0.06	14.0	0.0	[37]
5	Custard apple	30	0.71	18	21	382	4	-	-	-		19.2	0.08	0.10	0.50	0.14	0.22	-	2.0	[37]
6	Sugar apple	24	0.6	21	32	247	9	0.10	0.09		0.6	36.3	0.11	0.11	0.88	0.23	0.20	14.0	0.0	[37]
8	Nance	46	0.38	20	10	244	3	0.09	0.04	0.25	0.4	92.5	0.02	0.02	0.29	0.18	0.02	8.0	4.0	[37]
9	Bell pepper	9	0.37	11	22	188	3	0.17	0.05	-	0	97	0.06	0.05	0.66	-	0.25	23.0	67.0	[37]
10	Jalapeño pepper	12	0.25	15	26	248	3	0.14	0.05	0.10	0.4	118.6	0.04	0.07	1.28	0.32	0.42	27.0	54.0	[37]
11	Poblano pepper	10	0.34	10	20	175	3	0.13	0.07	-	0	80.4	0.06	0.03	0.48	-	0.22	10.0	18.0	[37]
12	Serrano pepper	11	0.86	22	40	305	10	0.26	0.13	-	0.4	44.9	0.05	0.08	1.54	-	0.51	23.0	47.0	[37]
14	Chilaca pepper	40	4.00	-	23	340	-	-	-	-	0.04	178.2	0.08	0.06	1.00	-	-	-	16.0	[40,45]
15	Habanero pepper	18	2.44	-	26	-	-	-	-	-	-	94	0.11	0.16	0.71	-	-	-	-	[40]
17	Papaya	20	0.25	21	10	182	8	0.08	0.05	0.04	0.6	60.9	0.02	0.03	0.36	0.19	0.04	37.0	47.0	[40]
18	Purple cainito	34	2.20	-	19	-	-	-	-	-	-	12.8	0.10	0.03	0.64	-	-	-	-	[40]
	White cainito	25	0.94	-	15	-	-	-	-	-	-	19.0	0.03	0.04	0.66	-	-	-	2.0	[40]
19	Mexican hawthorn	94	1.56	-	33	-	-	-	-	-	-	73.8	0.04	0.05	0.43	-	-	-	-	[37]
20	Zucchini	21	0.79	33	93	459	3	0.83	0.10	0.20	0.3	34.1	0.04	0.04	0.71	0.37	0.14	20.0	25.0	[37]
21	Black zapote	27	2.48	12	29	193	12	0.10	-	-	-	28.7	0.00	0.02	0.26	-	-	14.0	3.0	[40]
22	Dragon fruit ^1^	5	0.75	-	15	-	-	-	-	-	-	25.8	0.11	0.13	0.37	-	-	-	0.0	[40]
23	Sapodilla	21	0.80	12	12	193	12	0.10	0.09	-	0.6	14.7	0.00	0.02	0.20	0.25	0.04	14.0	3.0	[37]
26	Prickly pear	56	0.30	85	24	220	5	0.12	0.08		0.6	14	0.01	0.06	0.46		0.06	6.0	2.0	[37]
28	Avocado	12	0.55	29	52	485	7	0.64	0.19	0.14	0.4	10	0.07	0.13	1.00	1.39	0.26	81.0	7.0	[37]
29	Tomatillo	7	0.62	20	39	268	1	0.22	0.08	0.15	0.5	11.7	0.04	0.04	1.85	0.15	0.06	7.0	6.0	[37]
30	Canistel	20	1.00	-	42	-	-	-	-	-	-	43	0.02	0.02	3.13	-	-	-	-	[42]
31	Mamey	18	0.78	11	26	454	7	0.19	0.21	0.20	-	23	0.01	0.12	1.43	0.40	0.72	7.0	7.0	[37]
33	Guava	18	0.26	22	40	417	2	0.23	0.23	0.15	0.6	228.3	0.07	0.04	1.08	0.45	0.11	49.0	31.0	[37]
34	Squash	17	0.34	12	18	125	2	0.74	0.12	0.19	0.2	7.7	0.03	0.03	0.47	0.25	0.08	93.0	0.0	[37]
35	Tomato	5	0.47	8	29	212	42	0.14	0.06	0.09	0.4	16	0.05	0.03	0.59	0.19	0.06	29.0	75.0	[37]
36	Yellow mombin	34	3.00		73	-	-	-	-	-	-	51	0.10	0.05	0.94	-	-	-	-	[42]
37	Red mombin	22	0.60	-	40	--	-	-	-	-	-	43	0.07	0.03	1.00	-	-	-	-	[42]
38	White pitaya ^2^	-	-	-	-	-	-	-	-	-	-	55	-	-	-	-	-	-	-	[43]
	Yelllow pitaya	-	-	-	-	-	-	-	-	-	-	44.5	-	-	-	-	-	-	-	[43]
	Purple Pitaya	-	-	-	-	-	-	-	-	-	-	41.8	-	-	-	-	-	-	-	[43]
	Red pitaya	-	-	-	-	-	-	-	-	-	-	35.5	-	-	-	-	-	-	-	[43]

^1^*Hylocereus undatus* (Haw.) Britton et Rose; ^2^ *Stenocereus stellatus* (Pfeiff.) Riccob; ^3^ Retinol Activity Equivalents (RAE).

**Table 4:** Phenolic profile, total phenolic compounds (mg/100 g fresh weight), antioxidant capacity and in vivo and in vitro bioactivity of Mesoamerican fruits.

	Fruit	Total Phenolics ^a^	Antioxidant Capacity	Phenolic Profile	Bioactivity	Ref
1	Red cashew apple	118–740	618 ^1^ 274 ^2^	**Phenolic acids:** ferulic, ellagic, caffeic, protocatechuic, gallic, gentisic, *p*-coumaric, salicylic and sinapic acid **Flavonoids:** 3-*O*-galactoside, 3-*O*-glucoside, 3-*O*-xylopyranoside, 3-O-arabinopyranoside, 3-O-arabinofuranoside, 3-O-rhamnoside of myricetin and quercetin **Anthocyanins**: 5-methylcyanidin 3-O-hexoside and hexosides of cyanidin, petunidin and peonidin **Tannins:** (−)-epigallocatechin, (−)-epigallocatechin-O-gallate and (−)-epicatechin-3-O-gallate	In vivo anti-diabetic, antioxidant, anti-obesity and anti-inflammatory activity In vitro antioxidant activity	[46,47,48,49,50,51,52]
	Yellow cashew apple	186–634	642 ^1^ 345 ^2^			
2	Cherimoya	125–683	879 ^1^ 230 ^2^ 867 ^3^	**Phenylethanoids:** hydroxytyrosol hexoside **Phenolic acids:** 4-O-caffeyolquinic acid, caffeic acid-O-hexoside and sinapic acid **Flavonoids:** catechin, epicatechin and quercetin-3-O-glucoronide. **Tannins:** procyanidin dimers, trimers and tetramers types A and B **Lignins**	In vitro antioxidant and anticancer activity	[53,54,55]
3	Annona/Ilama	129–246	675 ^1^ 358 ^2^	Not reported	In vitro antidiabetic and antioxidant activity	[38,49,56,57]
4	Soursop	236–577	1451 ^3^	**Phenolic acids:** p-coumaric, coumaric acid hexose, 5-caffeoylquinic, caffeic acid derivative and dicaffeoylquinic acid **Flavonoids:** dihydrokaempferol-hexoside	In vitro antioxidant activity	[58]
5	Custard apple	358	650 ^1^ 376 ^2^	Not reported	In vitro antioxidant activity	[49]
6	Green sugar apple	208	646 ^1^ 369 ^2^	**Phenolic acids:** gallic, protocatechuic, caffeic, p-coumaric, sinapic and ferulic acid **Flavonoids:** catechin, epicatechin and epigallocatechin gallate **Tannins:** procyanidin B2	In vivo antidiabetic and antioxidant activity In vitro antioxidant activity	[49,59,60,61]
	Purple sugar apple	82	656 ^1^ 358^2^			
7	Chagalapoli	1051	4450^1^	**Phenolic acids:** derivates of caffeic and p-coumaric acid (hydroxycinnamoyl compounds) **Flavonoids:** (+)-catechin, (−)-epicatechin, myricetin-O-hexoside, kaempferol di-deoxyhexosyl-hexoside, kaempferol di-deoxyhexosyl-hexoside, (epi)catechin-3-O-gallate, quercetin 3-O-rutinoside and isorhamnetin rutinoside **Anthocyanins:** delphinidin 3-O-galactoside, petunidin 3-O-galactoside, cyanidin 3-O-galactoside, peonidin 3-O-galactoside and malvidin 3-O-galactoside **Tannins:** procyanidin B2	In vitro antioxidant activity	[62]
8	Green nance	195	669 ^1^ 381 ^2^	**Phenolic acids:** gallic, tetragalloylquinic, ellagic acid galloyl hexoside, protocatechuic, p-hydroxybenzoic, caffeic and p-coumaric acid **Flavonoids:** (−) epicatechin, catechin, rutin, taxifolin, quercetin pentoside, kaempferol, hesperidin, quercetin-3-O-xyloside, quercetin and quercetin-3-glucoside **Anthocyanins:** cyanidin-3-glucoside, pelargonidin-3-glucoside, peonin-3-glucoside and delphinidin-3-glucoside **Tannins:** proanthocyanidin dimers	In vivo antidepressant activity In vitro antioxidant activity	[49,63,64,65]
	Red nance	266	662 ^1^ 376^2^			
	Yellow nance	241	662 ^1^ 373 ^2^			
9	Green bell pepper	48–120	856–1717 ^1^ 228–560 ^2^ 399 ^4^	**Stilbenoids:** resveratrol Phenolic acids: gallic, caffeic and chlorogenic **Flavonoids:** myricetin, quercetin, quercetin 3-rutinoside, quercetin-D-glucoside, luteolin and kaempferol **Lignan amides:** p-aminobenzaldehyde, N-cis-feruloyl tyramine, N-trans-feruloyl tyramine, grossamide, N-trans-p-coumaroyl tyramine, N-trans-feruloyl octopamine and N-trans-p-coumaroyl octopamine **Phenolic amides:** dihydrocapsaicin	In vitro antibacterial, anti-inflammatory and antioxidant activity	[66,67,68,69]
	Red bell pepper	64–414	696 ^1^ 632^2^			
	Yellow bell pepper	55–260	504 ^1^ 472 ^2^			
10	Jalapeño pepper	92–244	229–538 ^2^ 4368–12, 420 ^3^ 55–659 ^4^	**Phenolic compounds in peppers (10–16):** **Phenolic acids:** sinapic acid-O-hexoside, caffeic acid glycoside, p-hydroxybenzoic acid β-glucoside and vanillic acid 1-O-β-D-glucopyranoside **Flavonoids:** quercetin, luteolin, kaempferol, apigenin, quercetin dihexoside, quercetin 3,7-di-O-rhamnopyranoside, apigenin apiofuranosyl-glucopyranoside, quercetin glucopyranoside, luteloin-glucopyranoside, naringenin chalcone hexose and naringenin 7-O-glucoside **Phenolic amides:** capsaicin, dihydrocapsaicin and nordihydrocapsaicin **Lignans:** Lariciresinol glucopyranoside	**Bioactivity in peppers (10–16):** In vivo antidiabetic, hypocholesterolemic, cardioprotective and antiobesity activity In vitro antidiabetic, anti-inflammatory, anticancer and antioxidant activity	[69,70,71,72,73,74,75,76,77,78]
11	Poblano pepper	188–305	48 ^2^ 0.5 ^5^ 62 ^6^			
12	Serrano pepper	69–296	242–476 ^2^ 6344–6844 ^3^ 487–555^4^	See section above (compilation of phenolics and bioactivity in peppers 10–16)		
13	Yahualica pepper	180	70 ^6^	See section above (compilation of phenolics and bioactivity in peppers 10–16)		
14	Chilaca pepper	974	710 ^1^ 47–55 ^2^ 215 ^4^	See section above (compilation of phenolics and bioactivity in peppers 10–16)		
15	Habanero pepper	16–232	2027–2694 ^1^ 260 ^2^ 481–898 ^4^	See section above (compilation of phenolics and bioactivity in peppers 10–16)		
16	Manzano pepper	132	^2^ 8900	See section above (compilation of phenolics and bioactivity in peppers 10–16)		
17	Papaya	45–159	661 ^1^ 270–988 ^3^	**Phenolic acids:** caffeic acid-O-hexoside-O-rhamnoside, caffeic acid hexoside-O-pentoside, protocatechuic acid-O-hexoside, ferulic and p-coumaric acid **Flavonoids:** quercetin-3-O(2′rhamnosyl)-rutinoside, quercetin-3-O-glucuronide and apigenin-O-pentoside	In vitro antiproliferative, anti-inflammatory and antioxidant activity	[53,58,79,80]
18	Green cainito	18–20	685 ^1^ 333 ^2^	**Phenolic acids:** gallic acid **Flavonoids:** (+)-catechin, (−)-epicatechin, (+)-galocatechin, (−)-epigallocatechin, quercetin, quercitrin, isoquercitrin and myricitrin **Tannins**	In vivo hypertensive and gastroprotective activity Ex vivo antihypertensive activity In vitro antihypertensive, anticancer and antioxidant activity	[49,81,82,83,84]
	Purple cainito	15–80	650 ^1^ 367 ^2^			
19	Mexican hawthorn	50–550	1472 ^5^ 0.06–0.35 ^6^	**Phenolic acids:** chlorogenic acid **Flavonoids:** (+)-catechin, (−)-epicatechin, rutin, vitexin, hyperoside, quercetin and vitexin 2-O-rhamnoside **Tannins:** procyanidin dimer, procyanidin trimer and progyadinidin tetramer	In vitro antioxidant and relaxant activity	[85,86]
20	Zucchini	519–867	12 ^6^ 370 ^5^	**Phenolic acids:** p-coumaric, ferulic, caftaric, chlorogenic, caffeic, 2-O-caffeoylmalic, chicoric, dicaffeic, sinapic acid hexoside, protocatechuic, p-hydroxybenzoic, benzoic, vanillic, vanillic acid glycoside and hydroxybenzoic acid hexose **Flavonoids:** quercetin 3-O-rhamnosyl-rhamnosyl-glucoside, luteolin O-glucoside, quercetin, isorhamentin, robinin, quercetin 3-rutinoside, quercetin O-glucoside, isorhamnetin O-rutinoside, kampeferol rutinoside, kaempferol O-glycoside, astragalin, myricetin and rutin **Tannins**	In vitro antioxidant activity	[87,88,89]
21	Black zapote	158–247	560 ^1^ 118 ^2^	**Phenolic acids:** cinnamic acid, p-hydroxybenzoic acid, dicoumaroylhexose-deoxyhexose, caffeic acid, sinapic acid, ferulic acid, o-coumaric acid and protocatechuic acid **Flavonoids:** catechin, epicatechin, myricetin, diapigenin hexoside, isorhamnetin hexose-malonate and dimyricetin hexose-malonate **Tannins**	In vitro antioxidant and anticancer activity	[49,90,91]
22	Dragon fruit	42–59	220–900 ^1^ 199 ^2^ 953 ^3^	**Phenylethanoid:** tyrosol **Stilbene:** coumarin **Phenolic acids:** gallic, ellagic, caffeoyl hexoside and p-coumaroyl quinic acid **Flavonoids:** quercetin 3-O-rutinoside, kaempferol hexoside, isorhamnetin hexoside, isorhamnetin 3-O-glucoside, eriodictyol hexoside, eriodictyol, naringenin acetylhexoside and taxifolin acetylhexoside **Tannins**	In vivo antidiabetic, wound healing and antihypertensive activity In vitro anticancer, anti-inflammatory and antioxidant activity	[49,58,79,92,93,94,95,96,97]
23	Sapodilla	15–159	405 ^1^ 208 ^2^ 4847 ^3^	**Phenolic acids:** 4-O-galloylchlorogenic, gallic, 4-O-galloylchlorogenate and methyl chlorogenate acid **Flavonoids:** quercitrin, myricitrin, (+)-catechin and (+)-gallocatechin	In vivo antitumor, anti-obesity, and antidiabetic activity In vitro antioxidant activity	[49,58,98,99]
24	Mamoncillo	295–647	665 ^1^ 322 ^2^	**Stilbenes:** resveratrol derivative **Phenolic acid derivatives:** p-coumaric acid derivative, caffeic acid derivative, ferulic acid derivative p-hydroxybenzoylhexose and p-coumaroylhexose acid	In vitro antioxidant activity	[49,100]
25	Cactus berry	740–1046	17 ^1^ 171 ^2^ 320 ^3^ 47–3300 ^4^	**Phenolic acids:** caffeic, gallic, vanillin, ellagic, protocatechuic, p-hydroxybenzoic, quinic and ferulic acid hexoside **Flavonoids:** quercetin, (−)-epicatechin, epigallocatechin, queretin-3-O-rhamnosyl rutinoside-glucoside, kaempferol-7-O-neohesperiodoside and isorhamnetin rhamnosyl-rutinoside **Tannins:** proanthocyanidins	In vivo antidiabetic and renal protective activity In vitro antioxidant, anti-inflammatory, antidiabetic and anticancer activity	[101,102,103,104]
26	Green prickly pear	38–62	2630 ^3^	**Phenolic acids:** piscidic, caffeic, ferulic, hydroxybenzoic, eucomic, protocatechuic, malic and succinic acid **Flavonoids:** isorhamnetin glucosyl-rhamnosyl-rhamnoside, isorhamnetin glucosyl-rhamnosyl-penstoside, isorhamnetin-hexosyl-hexosyl-pentoside, isorhamnetin glucosyl-pentoside, rutin, kaempferol-glucosyl-rhamnoside, isorhamnetin glucosyl-rhamnoside, isorhamnetin and isorhamnetin-3-O-robinobioside	In vivo antidiabetic, antioxidant and kidney protective activity In vitro anticancer, antioxidant, anti-inflammatory and antidiabetic activity.	[91,105,106,107,108,109,110]
	Purple prickly pear	282–350	308–630 ^2^ 2348–2378 ^3^			
	Red prickly pear	198–218	83–540 ^2^ 1988–2348 ^3^			
	Yellow prickly pear	62–158	23–345 ^2^ 1253–2115 ^3^			
27	Sour prickly pear	132–260	6400 ^1^ 988 ^2^ 42 ^6^ 253–313 ^7^	**Phenolic acids:** gallic, vanillic, 4-hydroxybenzoic, syringic, ferulic and protocatechuic acid **Flavonoids:** epicatechin, catechin, rutin, vanillin, quercetin, quercitrin and kaempferol	In vivo antidiabetic and antioxidant activity In vitro antioxidant activity	[111,112,113,114,115,116]
28	Avocado	11–490	130 ^2^ 1160 ^3^	**Phenylethanoids:** tyrosol-hexoside pentoside **Phenolic acids:** caffeic acid, α-resorcyclic acid, protocatechuic acid, p-coumaric acid glycoside, 5-feruloylquinic acid, ferulic acid, benzoic acid, trans-cinnamic acid, chlorogenic acid and sinapinic acid **Flavonoids:** catechin, epicatechin, epigallocatechin, rutin, quercetin, myricetin, kaempferol and isorhamnetin **Proanthocyanidins:** (epi)gallocatechin benzylthioether, catechin benzynthioether, epicatechin, benzylthioether and (epi)afzelchin benzylthioether and benzyl mercaptan	In vivo anti-obesity and antidiabetic activity In vitro anticancer, anti-inflammatory, antidiabetic, and antioxidant activity	[79,117,118,119,120,121,122,123]
29	Tomatillo	78–970	15–90 ^6^	**Phenolic acids:** chlorogenic, caffeoyl hexoside, coumaroyl hexoside, coumaroyl dihexoside, feruloyl dihexoside, sinapoyl hexoside and cinnamoyl dihexoside acid **Flavonoids:** quercetin, epicatechin, kaemperol-3-O-glycoside, quercetin-3-O-glycoside and dihydroflavonol **Anthocyanins** (in purple varieties)	In vitro antioxidant activity	[124,125,126]
30	Canistel	98	54 ^5^	**Phenolic acids:** gallic acid **Flavonoids:** (+)-gallocatechin, (+)-catechin and myricitrin **Tannins**	In vivo hepatoprotective and antioxidant activity In vitro antioxidant and anti-inflammatory activity	[127,128,129]
31	Mamey	14–29	394 ^1^ 113 ^2^	**Phenolic acids:** gallic, syringic, p-coumaric, protocatechuic hexose-malonate, hydroxybenzoic acid derivative, p-hydroxybenzoic acid dimer and p-hydroxybenzoic acid **Flavonoids:** epicatechin dimer, epicatechin, gallocatechin and catechin 3-O-gallate **Proanthocyanidins**	In vitro antioxidant activity	[49,130]
32	Capulin	243–331	130 ^2^	**Phenolic acids:** chlorogenic acid **Flavonoids:** (+)-catechin, quercetin hexoside, quercetin dipentoside, kaempferol hexoside, quercetin 3-O-glucoronide, rutin and quercetin-3-O-arabinoside **Anthocyanins:** cyanidin-3-O-glucoside and cyanidin-3-O-rutinoside and cyanidin **Proanthocyanidins:** procyanidin dimer B and procyanidin trimer B	In vivo antihypertensive and vasorelaxant activity In vitro antioxidant activity	[54,131,132,133]
33	Guava	175–462	3 ^1^ 1–300 ^2^ 1305 ^3^	**Phenolic acids:** gallic, chlorogenic, caffeic, p-coumaric, syringic, vanilic, ferulic and ellagic acid **Flavonoids:** catechin gallate, quercetin hexoside, quercetin pentoside, quercetin, (+) catequin, rutin and kaempferol **Proanthocyanidins:** PAC B-Type (E)GC-(E)C and PAC B-Type (E)Cg-(E)GC **Ellagitannins:** bilactone of valoneic acid	In vitro anticancer and antioxidant activity	[54,58,134,135]
34	Squash	80–494	76 ^2^ 34 ^4^	**Phenolic acids:** cinnamic, protocatechuic acid hexoside and coumaric acid **Flavonoids:** apigenin glucoside pentoside, luteolin-7-O-rutinoside, lutein-7-O-glucoside, muricitrin, apigenin 7-O-rutinoside, chrysoeriol 7-O-rutinoside, diosmetin and luteolin	In vivo antihypertensive, cardioprotective, antidiabetic Antiulcer and hepatic injury protective activity In vitro antioxidant activity	[136,137,138,139,140,141,142,143]
35	Tomato	11–142	80 ^2^ 10–27 ^6^	**Phenolic acids:** chlorogenic, caffeic, p-coumaric, ferulic, hydroxybenzoic acid hexose, protocatechuic, gentisic, dihydroxybenzoic acid pentose, benzoic acid, coumaric acid hexose, sinapic acid hexose, feruloylquinic, isoferulic, dicaffeoylquinic, caffeoyl-hexose, coumaroyl-hexose and tricaffeoylquinic acid **Flavonoids:** quercetin, kaempferol, naringenin, naringenin dihexose, rutin hexoside, apigenin acetylhexoside, quercetin 3,7-dihexoside, rutin pentoside, kaempferol 3,7-dihexoside, isorhamnetin 3-sophoroside, rutin, kaempferol-3-rutinoside and naringenin chalcone	In vitro anticancer and antioxidant activity	[55,91,144,145]
36	Yellow mombin	131–260	625 ^1^ 349 ^2^	**Flavonoids:** epicatechin and quercetin	In vivo gastroprotective and ulcer healing activity In vitro antioxidant activity	[49,146,147,148]
37	Red mombin	116–249	663 ^1^ 170–333 ^2^	**Phenolic acids:** 3-caffeoylquinic, dihydroxybenzoic acid hexoside and gallic acid **Flavonoids:** quercetin 3-O-pentosylhexoside, quercetin-3-O-pentosylrutinoside, quercetin pentoside, quercetin deoxyhexoside, rutin, quercetin-3-O-glucopyranoside, kaempferol-3-O-rutinoside, kaempferol-3-O-hexosyl-pentoside, astragalin and rhamnetin hexosyl pentoside	In vivo and in vitro antioxidant activity	[49,54,149]
38	Pitaya	18–160	250–900 ^1^ 2268–3369 ^8^	**Phenylethanoids**: tyrosol **Phenolic acids:** caffeoyl hexoside, feruloyl dihexoside and p-coumaroyl quinic acid **Flavonoids:** quercetin 3-O-rutinoside, kaempferol hexoside, isorhamentin hexoside, isorhamnetin 3-O-glucoside, eriodictyol hexoside, eriodictyol acetylhexoside, naringenin acetylhexoside and taxifolin acetylhexoside	In vitro antioxidant activity	[150,151]

^a^ mg/100 g fresh weight; ^1^ ABTS assay (µm TE/100 g fresh weight); ^2^ DPPH assay (µm TE/100 g fresh weight); ^3^ ORAC assay (µm TE/100 g fresh weight); ^4^ TEAC assay (µm TE/100 g fresh weight); ^5^ DPPH assay (IC_50_ µg/mL); ^6^ DPPH assay (scavenging activity %); ^7^ DPPH assay (mg quercetin equivalents/100 g fresh weight); ^8^ ABTS assay (mg Trolox equivalents/100 g fresh weight).

**Table 5:** Effects of High Hydrostatic Pressure (HHP) on phenolic compounds and parameters related to their stability in Mesoamerican fruits.

Fruit	Intensity (MPa)	Parameters Related to the Stability of Phenolic Compounds	Effect on Phenolic Content	Ref
Avocado slices	200	- Cell viability was retained. - Color was retained.	Not reported.	[163]
	>300	- Respiration rate and ethylene production ↓ 1 h after treatment and after 17 h at 20 °C. - Large oil droplets and internal disruption of cell walls.	Not reported.	
Avocado puree	600	- Antioxidant capacity ↓ post treatment but ↑ after 5 days of storage at 25 °C. - PPO and LOX activity ↓ due to HHP but regained activities at 10 to 15 days of storage at 4 °C.	Degradation (processing) Enhanced extractability (storage).	[164,165]
	345–689	- Low pH (3.9–4.3) contributed to microbiological safety. - ↓PPO activity and ↓ color change during 100 days of storage at 5 °C.	Not reported.	[166]
Bell pepper slices	100	- Greater microstructural damage to cells. - Antioxidant capacity ↓ 13%.	Degradation (processing).	[167]
	500	- Microstructure was retained. - Antioxidant capacity was retained. - Achieved microbiological safety.	Retention (processing).	
Cashew apple juice	250–400	- Soluble polyphenol content ↑ 25%. - Antioxidant capacity ↑ 45%. - Hydrolysable phenolics were retained.	Enhanced extractability (processing).	[168]
Guava puree	400	- Inactivation of POD, PPO and PME post treatment. - After 60 days of storage, higher enzymatic activity.	Not reported.	[169]
	600	- PPO and pectinesterase activity ↓ post-treatment. - After 60 days storage, POD and PME was similar to control and PPO was higher than the control.	Not reported.	
Papaya beverage	550	- Achieved microbiological safety. - Phenolic content ↓ 11% post treatment - Phenolics were retained during storage (40 days at 4 °C). - Antioxidant capacity ↓ 9% post treatment. - Antioxidant capacity was retained during storage.	Degradation (processing) Retention (storage).	[170]
Pitaya beverage	400	- Phenolic content ↓ 20%.	Degradation (processing).	[171]
	550–600	- Phenolic compounds were retained post-treatment and during storage (60 days at 4 °C). - Antioxidant capacity was retained post-treatment and during storage. - Reduction of PME activity.	Retention (processing and storage).	[172]
	600	- Phenolic content ↓ 15% post treatment. - Reduction of PME activity.	Degradation (processing).	[171]
Prickly pear beverage	550	- Total phenolics ↑ 35%. - Antioxidant activity ↑ 13%. - Kaempferol and isorhamnetin were retained.	Enhanced extractability (processing).	[173]
Prickly pear slices	100	- Higher phenolic acid content: piscidic acid ↑ 30% and hydroxybenzoic acid ↑ 70%. - Flavonoid content ↑ 11%. - Antioxidant capacity ↑ 41%. - Anti-inflammatory activity ↑ 25%. - Damaged cell walls, plasmodesma and tonoplast.	Enhanced extractability (processing).	[174,175]
	350	- Higher phenolic acid content: piscidic acid ↑ 40% and hydroxybenzoic acid ↑ 75%. - Flavonoid content ↑ 135%. - Antioxidant capacity ↑ 81%. - Anti-inflammatory activity ↑ 41%. - Ruptured cell membrane, cell walls and tonoplast.	Enhanced extractability (processing).	[174,175]
	600	- Higher phenolic acid content: piscidic acid ↑ 50% and hydroxybenzoic acid ↑ 120%. - Flavonoid content ↑ 141%. - Antioxidant capacity ↑ 62%. - Anti-inflammatory activity ↑ 86%. - Severe damage to cells.	Enhanced extractability (processing).	[174,175]
	400–600	- Phenolic content ↑ 20%.	Enhanced extractability (processing)	[176]
Sapodilla jam	400	- Phenolic content ↑ 27%.	Enhanced extractability (processing).	[177]
Tomato juice	250	- Antioxidant capacity was retained post treatment and during storage (30 days at 25 °C).	Retention (processing and storage).	[178]

**Table 6:** Effects of other innovative technologies on phenolic compounds and parameters related to their stability in Mesoamerican fruits.

Fruit	Intensity	Parameters Related to the Stability of Phenolic Compounds	Effects on Phenolic Content	Ref
Pulsed Electric Fields				
Tomato juice	20 kV/cm	- Highest antioxidant capacity (depended on electric field strength and treatment time).	Not reported.	[193]
	35 kV/cm	- Retained phenolic content and antioxidant capacity post treatment and during storage (91 days at 4 °C).	Retention (processing and storage).	[194]
Tomato fruit	1 kV/cm	- Phenolic compounds ↑ 19% after 24 h at 4 °C. - Chlorogenic acid ↑ 25%. - Ferulic-O-glucoside acid was retained. - Caffeic-O-glucoside acid ↑ 17%.	Synthesis (storage).	[181]
	1.2 kV/cm	- Total phenolics ↑ 57% after 24 h at 4 °C. - Hydroxycinnamic acids (chlorogenic acid ↑ 152%, caffeic acid-O-glucoside ↑ 170%, caffeic acid ↑ 140%), - Flavanones (naringenin ↑ 15%, naringenin-7-O-glucoside ↑ 67% and eridictyol ↑ 5%). - Retention of flavonols, coumaric and ferulic acid-O-glucoside.	Synthesis (storage).	[182]
	1.2 kV/cm	- Total phenolics ↑ 44% after 24 h at 4 °C.	Synthesis (storage).	[195]
**Ultrasound**				
Avocado puree	20 kHz	- PPO activity increased. - Higher viscosity. - Decrease in particle size and disruption of structure.	Not reported.	[196]
Cashew apple puree	226 W/cm^2^	- Disruption of suspended fibers. - Highest phenolic content and antioxidant capacity (depended highly on the bagasse: water ratio).	Enhanced extractability (processing).	[197]
Custard apple juice	20 kHz	- Phenolic content ↑ 15%. - No effect on °Brix. - Inactivation of peroxidase and PME.	Enhanced extractability (processing).	[198]
Guava juice	20 kHz+cellulase	- 21% higher extraction yield. - Higher °Brix. - Phenolic content ↑ 16%. - Antioxidant capacity ↑ 12% to 20%.	Enhanced extractability (processing).	[199]
Prickly pear juice	20 kHz	- Assured microbiological safety. - Higher °Brix. - Phenolic content ↑ 40%. - Antioxidant capacity ↑ 50%.	Enhanced extractability (processing).	[200]
Soursop puree	24 kHz 50 °C +vacuum	- Microbial inactivation ≥ 7 CFU of *E. coli* and *S. aureus*. - PPO activity ↓ 94%. - No changes in sensory attributes.	Not reported.	[201]
Tomato fruit	45 kHz	- Total phenolic compounds ↑ 40% after 15 days at 10 °C (greatly influenced by storage time). - Reduction of microbiological load.	Synthesis (storage).	[187]
Tomato beverage	37 kHz	- Retention of phenolic compounds.	Retention (processing).	[202]
**Microwave**				
Avocado puree	11 W/g	- PPO ↓ 80% and was retained during storage. - PME activity was not detected post-treatment. - Phenolic content ↑ 29% post treatment and was retained (4 weeks at 4 °C). - Increase in viscosity due to release of soluble pectin.	Enhanced extractability (processing).	[191]
Guava nectar	500–950 W	- Inactivation of PME. - Retention of ascorbic acid. - Microbial counts below detectable levels (12 days at 4 °C).	Not reported.	[203]
Jalapeño pepper	Not reported	- Phenolic compounds ↓ 21%. - Antioxidant capacity ↑ 45%.	Degradation (processing).	[192]
Mamey pulp	937 W	- The 165 s treatment completely inactivated PPO. - Retention of pulp microstructure.	Not reported.	[204]
**Cold Plasma**				
Cashew apple juice	nitrogen 80 kHz	- Flavonoids ↑ 120% and polyphenols ↑ 128%. - Ascorbic acid content ↑ 11%. - Antioxidant capacity ↑ 130%. - Overexposure to plasma ↓ most bioactive compounds.	Enhanced extractability (processing).	[205]
Pitaya fruit	60 kV	- Total phenolic content ↑ 28% post treatment. - Antioxidant capacity ↑ 21% post treatment. - Gallic acid ↑ 107%, protocatechuic acid ↑ 132% and p-coumaric acid ↑ 109% after 36 h storage at 15 °C. - Cutting ↑ phenolic content and antioxidant activity in control group, while cold plasma further ↑ these values. - Cold plasma amplified signal role of ROS and activated phenylpropanoid metabolism.	Enhanced extractability (processing) Synthesis (storage).	[206]
Dragon fruit	argon 40 W	- Pathogen growth was inhibited (>15 days). - Phenolic compounds were retained.	Retention (processing).	[207]
Tomato beverage	50 kHz	- Phenolic content ↑ 5%.	Enhanced extractability (processing).	[202]
**Ultraviolet light**				
Pitaya juice	UV-C 57 µW/cm^2^	- Phenolic compounds were retained. - Reduction of 1.8 log cycles of *Z. bailii*.	Retention (processing).	[208]
Prickly pear fruit	UV-B 6.4 W/m^2^	- Phenolic compounds were retained post treatment. - After 24 h at 16 °C, phenolic compounds ↑ 100% in whole pulp and ↑ 25% in wounded pulp.	Retention (processing)Synthesis (storage).	[209]
Tomato beverage	UV-C Not reported	- Retention of phenolic compounds.	Retention (processing).	[202]

## Abbreviations

PPO	Polyphenol oxidase
PME	Pectin methyl esterase
POD	Peroxidase
HHP	High Hydrostatic Pressure
PEF	Pulsed Electric Fields
US	Ultrasound
MW	Microwave
CP	Cold Plasma
UV light	Ultraviolet light
